# Application of Nanoparticles Alleviates Heavy Metals Stress and Promotes Plant Growth: An Overview

**DOI:** 10.3390/nano11010026

**Published:** 2020-12-24

**Authors:** Pingfan Zhou, Muhammad Adeel, Noman Shakoor, Manlin Guo, Yi Hao, Imran Azeem, Mingshu Li, Mengyuan Liu, Yukui Rui

**Affiliations:** Beijing Key Laboratory of Farmland Soil Pollution Prevention and Remediation, College of Resources and Environmental Sciences, China Agricultural University, Beijing 100193, China; zhoupingfan0516@163.com (P.Z.); Chadeel969@gmail.com (M.A.); nomanshakoor1993@gmail.com (N.S.); mlguo0829@163.com (M.G.); hy0305hy@163.com (Y.H.); imranazeem18@gmail.com (I.A.); mingshuqy@163.com (M.L.); lmy00172913@163.com (M.L.)

**Keywords:** nanoparticles, plant stress, uptake and transport, combined application, heavy metals

## Abstract

Nanotechnology is playing a significant role in addressing a vast range of environmental challenges by providing innovative and effective solutions. Heavy metal (HM) contamination has gained considerable attention in recent years due their rapidly increasing concentrations in agricultural soil. Due to their unique physiochemical properties, nanoparticles (NPs) can be effectively applied for stress alleviation. In this review, we explore the current status of the literature regarding nano-enabled agriculture retrieved from the Web of Science databases and published from January 2010 to November 2020, with most of our sources spanning the past five years. We briefly discuss uptake and transport mechanisms, application methods (soil, hydroponic and foliar), exposure concentrations, and their impact on plant growth and development. The current literature contained sufficient information about NPs behavior in plants in the presence of pollutants, highlighting the alleviation mechanism to overcome the HM stress. Furthermore, we present a broad overview of recent advances regarding HM stress and the possible mechanism of interaction between NPs and HM in the agricultural system. Additionally, this review article will be supportive for the understanding of phytoremediation and micro-remediation of contaminated soils and also highlights the future research needs for the combined application of NPs in the soil for sustainable agriculture.

## 1. Introduction

Rapid urbanization and industrialization, constant application of agrochemicals and fertilizers, unreasonable mining and waste management have caused heavy metal (HM) contamination to become an increasingly prominent issue [1,2,3]. There are over 10 million contaminated sites worldwide and more than 50 percent of them are contaminated with HM [4]. According to the China Ministry of Environmental Protection, the percentage of exceedance in agricultural soils is even greater than 19.4%. Among them, the average contents of Cd, Hg, As, Cu, Pb, Cr, Zn and Ni in the soil exceed the standard by 7.0%, 1.6%, 2.7%, 2.1%, 1.5%, 1.1%, 0.9% and 4.8%, respectively [5]. Due to the Chinese diet structure, the risk screening values for soil contamination of agricultural land are low. For example, the China agricultural land Cd limit is 0.3~0.6 mg/kg, while the screening value (ECOSSL) recommended by the United States Environmental Protection Agency (US-EPA) is 0.4~0.8 mg/kg [3,6,7]. Only in China, the HM contaminated area of farmland soil is about 2 × 10^7^ Hm^2^, the annual amount of polluted grains is 1.2 × 10^7^ t and the economic loss is $3 × 10^9^ [8]. Recently, several studies have shown that HM pollution in farmland soil is continuously increasing [9,10].

In recent years, HM contamination has attracted public attention due to the frequent detection in food and blood. HM stress affects the plants growth and indirectly affects human health via the food chain [11]. Cereal, as a predominant food in China, are susceptible to HM pollution. Rice in particular accumulates more HM than other plants [12,13]. The notorious “Itai-Itai disease” in Japan is caused by long-term consumption of Cd-contaminated rice. Besides, excessive accumulation of HM causes systemic bone softening, nervous system damage, reproductive dysfunction and even death [14]. The Agency for Toxic Substances and Disease Registry (ATSDR) and the US-EPA have listed Cd, Pb, As and Hg among the top 20 hazardous substances. Furthermore, the current status of the rare earth elements (REEs) in the environment and potential effects on biota were described in our previous publication [15]. The deleterious effects of REEs and HM pollution in the soil environment require serious attention to overcome.

HM pollution is one of the key factors restricting crop production and threatening food security [16,17]. HM mainly affect the normal cell structure, the antioxidant system and plant growth to restrict crop production. More importantly, the global population is estimated to grow to 8.54 billion in 2030 and 9.73 billion in 2050, which means that current food production must increase by 70~100 percent to meet the needs of the world population [18,19]. Therefore, it is necessary to improve and popularize HM removal/immobilization technology in polluted farmland.

In recent years, the applications of nanoparticles (NPs) have increased dramatically in industry, medicine, agriculture and cosmetics fields [20]. NPs usually refer to materials with at least one dimension smaller than 100 nm. NPs with different particle sizes, geometries and functions can be synthesized according to the needs [21]. Compared with ordinary materials, NPs have many advantages, such as high surface activity, more surface reaction sites, good catalytic efficiency, and unique optical and magnetic properties [22,23,24]. The effects of NPs on environment have been discussed in our previous studies, such as Hao et al. demonstrated that fungal endophytes in rice were sensitive to carbon-based NMs even at 10 mg/L [25]. Rui et al. reported that biomass and quality of peanut were negatively influenced by exposure to Ag NPs at 50 mg/kg [26]. Additionally, Adeel et al. found that low doses (5 and 50 mg/kg) of NiO did not affect the survival, reproduction, and growth rate of adult earthworms, while high doses (200 and 500 mg/kg) significantly affected the physiological and biochemical endpoints [20].

NPs have been already successfully applied in agriculture and in environmental applications. Several studies have revealed that NPs could improve plant seed germination, plant photosynthesis, resistance to oxidative stress, rhizome growth and development, crop yield and quality [27,28,29,30]. On the one hand, NPs are applied as nanofertilizers [31,32,33] and nanopesticides [34,35,36,37], which have the advantages of being easily absorbed by plants and slowly released in the environment compared with traditional fertilizers [38], but the potential ecological risks still need further research [39]. On the other hand, NPs (such as CeO_2_, TiO_2_, and Mn_3_O_4_ NPs) can enhance the activity of antioxidant enzymes, which can reduce the accumulation of reactive oxygen species (ROS) in plants, alleviating plant stress and improving the quality and yield [40,41].

The development of nanoscience provides a new direction for the advancement of soil remediation [42]. Due to the unique properties, NPs not only have a better repair effect than traditional materials but also can add some new functions [43]. Except through soil, foliar spraying is another way to improve plants resistance to HM [40,44]. For example, selenium and silicon NPs alleviated Cd and Pb stress in rice through foliar application [45]. Foliar spraying of TiO_2_ NPs is more effective than soil addition in reducing Cd toxicity to maize [46].

Considering the worldwide high levels of contamination of HM, especially in China, this article will focus on HM stress management. Based on the published literature (January 2010 to November 2020), this review will discuss applying different NPs to plants under HM stress and the possible mechanisms of interaction between NPs and HM.

## 2. Analysis of Current Research Hotspots

### 2.1. Data Collection and Processing

Literature data was collected from the Web of Science (WoS) Core Collection database, regarded as the most comprehensive [47]. The search terms for the data were set as Topic: (Heavy metal contaminated soil or heavy metal stress or soil with heavy metal pollution) and Topic: (Plant or botany or crop) and Topic: (Nano or nanomaterials or nanoparticles or NPs). After screening according to the title and abstract, a total of 124 articles were eventually retrieved. We found that according to our retrieved documents, the first relevant literature appeared in 2010.

### 2.2. Scientometrics Analysis Methods

CiteSpace is a bibliometric software developed by Chen et al. used to analyze documents [48]. It can provide the functions of analyzing research status, finding research frontiers and hotspots, and promoting knowledge interaction between different fields [49]. Time-zone chart of high frequency keywords, keywords clustering and top 10 keywords with the strongest citation bursts were conducted in CiteSpace 5.6. R5.

### 2.3. Results and Discussion

**Figure 1:**
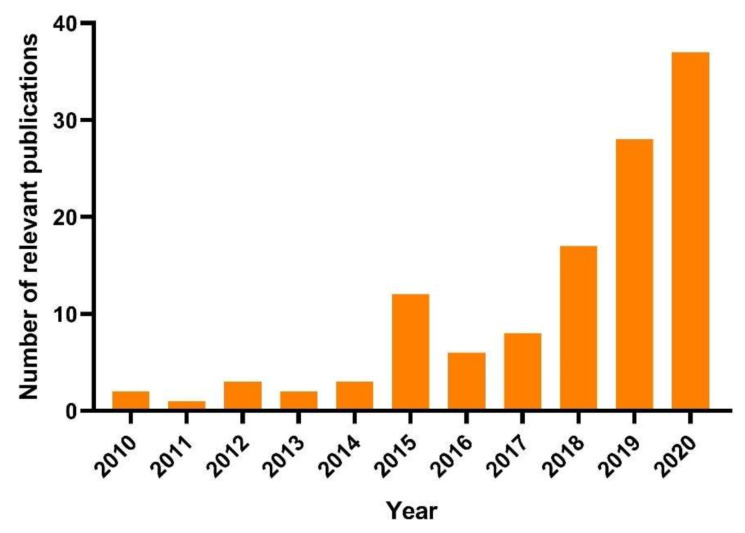
The number of relevant publications in the past ten years. Articles were collected from the Web of Science (WoS) Core Collection database. The search terms for the data were set as Topic: (Heavy metal contaminated soil or Heavy metal stress or Soil with heavy metal pollution) and Topic: (Plant or Botany or Crop) and Topic: (Nano or Nanomaterials or Nanoparticles or NPs).

**Figure 2:**
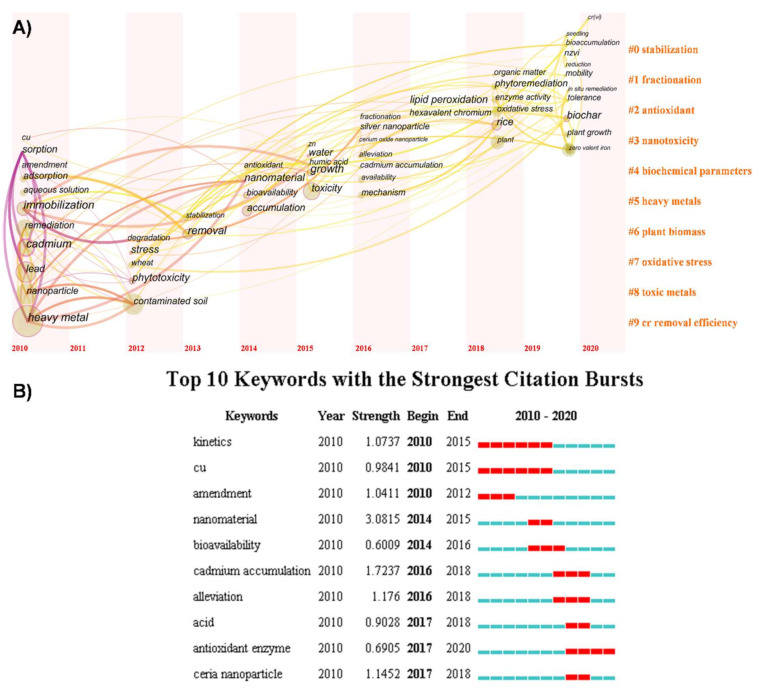
(**A**) Time-zone chart of high frequency keywords and clustering of keywords from January 2010 to November 2020. Node location indicates the time of keywords first appear and the size of node indicates the frequency of keyword occurrence in the studied documents. The orange font on the right shows the clustering results of keywords, indicating some research topics. (**B**) Top10 keywords with the strongest citation bursts. Strength represents the amount of a particular keyword being cited over a period of time, which indicates the most active area of research. Red bars indicate the start and end of a particular keyword citation burst.

The number of relevant articles have increased exponentially in the past ten years (Figure 1). Through the analysis of keywords, we can reveal the characteristics and development trends of this field. As shown in (Figure 2A), each circle represents a keyword that first appeared in the data set being analyzed. We can judge the research interest (frequency) according to the node’s size and judge the publication year based on the time zone [50]. Moreover, the clustering of keywords indicates some research topics, which can help us understand the structure of this field. Besides, top 10 keywords with the strongest citation bursts can be obtained according to the changes of citations over time (Figure 2B) [51]. All keywords with the strongest citation bursts indicate the most active area of research.

Based on the analysis of the data in Figure 2A,B, the literature regarding the NPs used to treat HM-contaminated soil emerged in 2010. In the early stage, the research mainly focused on the use of NPs for remediation of HM in the soil as an amendment and the adsorption kinetics of NPs. Literature regarding Cd first appeared in 2016, and the reason may be the high over-the-standard rate of Cd levels in China. In more recent literature, the focus has turned to HM bioavailability and HM accumulation in plants. In real environmental conditions, soil HM pollution is caused by a combination of several metals and not only Cd. Therefore, to get closer to the actual pollution situation, researchers began to study the composite pollution of HM in recent years. Later, interest in plant physiology and biochemistry have increased because some researchers found that the application of NPs may enhance plant antioxidant enzyme activity, thereby reducing plant oxidative stress damage caused by HM, promoting plant growth and reducing HM content in the edible parts of plants. Since 2017 CeO_2_ NPs have been extensively studied because CeO_2_ NPs can effectively reduce ROS levels and protect chloroplasts of the plant under stress due to Ce^3+^ and Ce^4+^ dangling bonds on the surface. In addition to the aforementioned topics, phytoremediation, biochar, and combined applications are emerging research areas, based on the findings that NPs may increase plant resistance to HM and significantly increase the plant’s ability to adsorb HM. Moreover, the research about the combined use of NPs with biochar, microorganisms or other materials is increasing rapidly in recent years, showing excellent application potential.

## 3. Nano-Remediation in Plant Under HM Stress

### 3.1. Mechanisms of Nanoparticles to Alleviate the HM Stress

**Figure 3:**
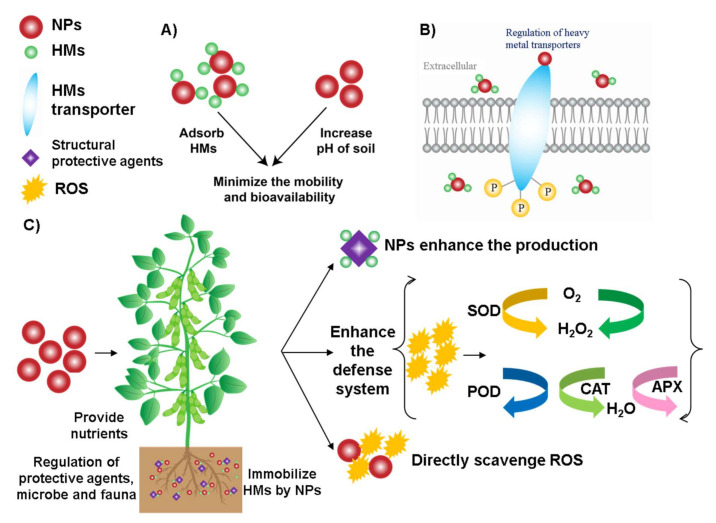
Mechanisms of NPs to alleviate HM stress in soil and plant tissues. (**A**) NPs increase the soil pH, adsorb HM, which minimize the mobility and bioavailability of HM. (**B**) NPs can enhance the defense ability of plants by regulating the HM transport genes. (**C**) NPs enhance the plant defense system, promote the production of protective agents and directly scavenge ROS, which ultimately improve the plant growth and nutritional content of fruits.

The morphology, physiology and biochemistry of the plant have been shown to be influenced by HM stress. There are several strategies applied to overcome the HM stress in plants (Figure 3). For example, (1) reducing the concentration of bioavailable HM in the soil [43], (2) regulating the expression of HM transport genes in plants [52], (3) enhancing the ability of plant antioxidant systems and improving the physiological functions [41] and (4) promoting the production of protective agents (such as root exudates, phytochelatin and organic acids) [46,52,53].

The HMs in the soil can be absorbed and transformed by the NPs, reducing the mobility and bioavailability of HM. For example, Fe_3_O_4_ NPs decreased the mobility of Cd and other HMs in the soil [54]. Wang et al. reported that mercapto Si NPs transformed Cd into a more stable constituent under field conditions after three years [55]. Besides, some NPs can improve soil properties, such as hydroxyapatite NPs can release phosphate and increase soil pH, reducing the harmful effects of HM in the soil [56].

Apoplastic barriers in the plant root have the physiological function of protecting the plant and controlling the flow of water, ions and oxygen [57]. The formation of apoplastic barriers can be influenced by NPs, which may decrease the amount of HM in the root [58]. However, the apoplastic barriers alone may not be an effective strategy to reduce the damage caused by HM stress because plant roots contain various ion and protein transporters in the root cell plasma membrane, which can transport HM simultaneously. Moreover, plant metal transport genes can be regulated by specific NPs, thereby enhancing the plant’s extracellular barrier to intercept HM.

Most NPs accumulate in the cell walls, bind with HM and make them unavailable by forming complexes. These complexes become adsorbed on the cell surface [59,60], thereby hindering the migration of HM in plants and reducing their biological activity. Besides, organic acids accumulated in the cell walls of plant roots and leaves can be chelated with HM to reduce the damage of HM stress to plants. The production of structural protective agents has been shown to be enhanced by NPs, such as applying exogenous Si NPs which promoted the synthesis of organic acids and reduced the damage of Cd to the plant.

Another strategy to alleviate HM stress is to activate the oxidation defense system of the plant. Plants usually produce ROS due to specific biochemical reactions [61]. For example, during the metabolic processes such as respiration and photosynthesis, plants continuously produce ROS in chloroplasts and other parts of cells. At low levels, ROS act as signal molecules involved in growth, development, and defense. However, under stress conditions, excessive ROS accumulation is harmful to cell membranes, proteins, and other cellular components [61]. ROS in plants is mainly scavenged by antioxidant enzymes, such as superoxide dismutase (SOD), catalase (CAT), ascorbate peroxidase (APX), glutathione reductase (GR), glutathione peroxidase (GPX) and peroxidase (POD). In addition, non-enzymatic low-molecular weight metabolites also can scavenge ROS, such as vitamin C, vitamin E and polyphenols [62,63,64]. Metabolic pathways related to ROS clearance will be triggered under stress. For example, shikimate-phenylpropanoid biosynthesis and galactose, alanine, aspartic acid and ascorbate metabolism can alleviate the oxidative stress of the plant [46,65]. Therefore, application of NPs with antioxidant enzyme activity (such as CeO_2_ NPs, Fe_3_O_4_ NPs, Mn_3_O_4_ NPs and C_60_) can enhance the ability of plants to reduce ROS, ultimately minimize the effects on crop growth and yield losses [61,66,67,68].

### 3.2. Impacts of Metal-Based Nanoparticles on the HM Stress in Plants

#### 3.2.1. Cerium Dioxide

CeO_2_ NPs are light rare earth elements (LREEs), recommended by the Organization for Economic Co-operation and Development (OECD) for safety evaluation compared to other ENPs due to their widespread applications and beneficial effects in agriculture systems [15,69]. CeO_2_ NPs to alleviate the stress is mainly due to the Ce^3+^ and Ce^4+^ dangling bonds on the surface that can scavenge ROS and minimize oxidative stress caused by HM [61,70]. For example, negatively charged CeO_2_ NPs effectively accumulated in chloroplasts and eliminated ROS in Arabidopsis leaf mesophyll cells compared with positively charged NPs, while the process was affected by the ratio of Ce^3+^/Ce^4+^ [61]. Several studies have indicated that the low concentration of CeO_2_ NPs is beneficial to the plant growth. For example, Cao et al. found that CeO_2_ NPs (100 mg/kg), both uncoated and polyvinylpyrrolidone (PVP) coated, can increase the photosynthesis rate of soybeans and stimulate plant growth, whereas 500 mg/kg CeO_2_-NPs reduced the net photosynthesis rate by 36% [71]. CeO_2_ NPs (100 and 200 mg/kg) also improved the physiological parameters of *Brassica napus* L. under salt stress (100 mM NaCl) [72]. Ma et al. determined that bulk CeO_2_ (10 and 100 mg/L) increased biomass of *Brassica rapa*, while at the same concentration, CeO_2_ NPs exerted no significant effect [73]. This might happen due to the formation of aggregates and the selected concentration of CeO_2_ NPs. These studies indicate that there are still many unknowns regarding the effect of NPs surface modifications, which strongly affect the stability and mobility of NPs, on the plant health.

Foliar application of CeO_2_ NPs (200 mg/L) effectively stimulated the antioxidant defense system in rice grown in hydroponic conditions (CdCl_2_ 50 μM) and inhibited the accumulation of Cd [69]. Furthermore, CeO_2_ NPs (500 mg/kg) did not affect the accumulation of Cd in soybeans in Cd-contaminated soil (0.2542 and 1.0 mg/kg) while significantly increased the accumulation of Ce in plant tissues [58]. This was partly caused by the co-exposure of Cd and CeO_2_ NPs that influenced root apoplastic barriers. Wang et al. reported that CeO_2_ NPs (100 mg/L) did not affect the accumulation of As (III) (1.0 mg/L) and As (V) (1.0 mg/L) in rice in hydroponic conditions [74]. As mentioned above, several contradictory studies are reported, and more in-depth investigations are needed to understand the alleviation mechanisms.

#### 3.2.2. Titanium Dioxide Nanoparticles

Titanium dioxide NPs (TiO_2_ NPs), widely used in coatings, cosmetics and pigments, are among the most produced engineered NPs in the world due to the low toxicity, favorable optical properties, strong adhesive properties and high stability [75]. It is estimated that the global annual production of TiO_2_ NPs was 1,175,176 tons in 2012 [76,77]. The primary mechanisms of TiO_2_ NPs in alleviating HM stress include adsorption of HM and activation of the oxidation defense system. Several studies have found that application of TiO_2_ NPs can reduce the oxidative stress of plants. For example, Wang et al. found that adding TiO_2_ NPs to the nutrient solution increased antioxidant enzymes’ activity in corn tissues [78]. Singh et al. reported that the physiological parameters and photosynthetic rate of soybean increased after applying TiO_2_ NPs to soil, effectively limiting the toxicity of Cd to soybean plants [79]. Therefore, TiO_2_ NPs have great application potential in alleviating plant oxidative stress caused by HM.

Cai et al. documented that accumulation of Pb in rice co-exposed to Pb(NO_3_)_2_ (1.0 mg/L) and four types of TiO_2_ NPs (10 and 1000 mg/L) [80]. They found that none of the TiO_2_ NPs affected rice growth, but three types of NPs (anatase, pristine rutile and rutile with hydrophilic) effectively decreased the Pb accumulation in roots by >80% and in shoots by 77~97%. The same results were obtained by Ji et al., who showed that TiO_2_ NPs had little effect on rice seedling biomass in hydroponic cultivation [81]. However, the root length, plant height, hormone level, antioxidant enzyme activity and other physiological parameters were improved after exposure of TiO_2_ NPs, indicating that the addition of TiO_2_ NPs reduced the damage of Cd stress to rice seedlings.

Maize was grown in Cd-contaminated soil then TiO_2_ NPs (100 and 250 mg/L) were applied by mixing in soil and by foliar application [46]. The results showed that foliar application of TiO_2_ NPs inhibited Cd absorption by maize and increased biomass, while soil application promoted the absorption of Cd by maize and substantially decreased the biomass. They also found that foliar spraying of TiO_2_ NPs increased SOD and GST activities and up-regulated galactose, alanine, aspartame acid and other metabolic pathways to alleviate Cd stress damage to maize. The results indicated that the application method had a significant influence on the alleviation effect.

Dai et al. studied the effect of uncoated TiO_2_ NPs and TiO_2_ NPs coated with sodium dodecylbenzene sulfonate (SDBS) on wheat seedlings under Cd stress [82]. After SDBS coating, the hydrodynamic diameter of TiO_2_ NPs was reduced, and the dispersion stability of TiO_2_ NPs was improved through spatial and electrostatic repulsion, thereby exposing more available adsorption sites and increasing the adsorption capacity of Cd^2+^. They also found that the effect of SDBS coated TiO_2_ NPs on relieving Cd toxicity were significantly better than these of bare TiO_2_ NPs. This suggests that NPs coated with surfactants or other materials affect the bioavailability and toxicity of HM.

Singh et al. reported that the accumulation of ^133^Cs in soybean effectively increased after the application of TiO_2_ NPs in ^133^Cs contaminated soil [83]. It suggests that NPs may be applied for phytoremediation, but long-term field-based studies are needed.

#### 3.2.3. Iron

Iron (Fe) is an essential element for humans, animals and plants’ growth and development [33]. It plays a vital role in physiological and biochemical reactions, such as cell metabolism, photosynthesis, and respiration [84]. For example, the synthesis of specific chlorophyll-protein complexes in chloroplasts requires Fe, and Fe deficiency causes yellowing leaves and reduced photosynthetic capacity [85]. Iron is also a cofactor for certain enzymes, closely related to enzyme activity [86]. Iron NPs (Fe NPs) are particularly powerful adsorbents due to the unique structure and electronic properties. Magnetic NPs (γ-Fe_2_O_3_ and Fe_3_O_4_) can be easily separated from the adsorbing medium by a magnetic field [84]. The alleviation mechanisms of nano Fe mainly include adsorption of HM, acting as an essential element, promotion of the formation of root surface iron film, activation of the oxidation defense system, and scavenging the ROS [66]. Several studies have demonstrated that nano Fe has high application potential in the field of fertilizers. For example, Fe_2_O_3_ NPs have been proposed as Fe-containing fertilizers in the cultivation of peanuts, but the potential risks still need to be fully elucidated [31]. In addition, it has been shown that legume root growthwas enhanced by 88~366% after using low dose Fe_2_O_3_ NPs pre-soaking [29].

Nanoscale zero-valent iron (nZVI) has been shown to reduce the accumulation of HM in sunflowers, increase SOD and POD in plant leaves, and promote plant growth [87]. Guha reported that after applying nZVI (100 mg/L) to rice seedlings, gene expressions of Fe transporters (IRT1, IRT2, YSL2, YSL15) responsible for both Fe and Cd uptake were significantly down-regulated. In contrast, OsVIT1 and OsCAX4 genes were overexpressed, which lead to sequestration of Cd in vacuoles [88].

Hussain et al. found that soil and foliar application of Fe_2_O_3_ NPs (5, 10, 15, and 20 ppm) to wheat under Cd stress (available Cd 0.93 mg/kg) both reduced the leaf electrolyte leakage rate, the Cd content in grains and increased the antioxidant enzyme activity and the dry weight of the wheat [89]. They also found that the foliar spraying of Fe NPs is preferred to the soil application because the absorption of Fe in the soil may be affected by many factors, such as pH, and the combination with other minerals during the absorption process. In the case of combined application, the alleviating effects of Fe NPs (foliar) application on Cd stress in rice were further enhanced when the soil was applied with biochar [90]. However, the biochar-Fe_3_O_4_ nanocomposites facilitated Cd transport in water-saturated natural soil, indicating that the risks are still not fully understood [91]. In composite stress conditions, drought stress increased the Cd concentration in grains, and the soil application of Fe_2_O_3_ NPs to wheat under drought and Cd stress improved the photosynthesis, yield and decreased the Cd content in grains [92].

#### 3.2.4. Zinc Oxide Nanoparticles

Zinc (Zn) is an essential trace element for plants and humans. Zinc oxide NPs (ZnO NPs) are common metal oxide NPs (MNPs), widely used in personal care products, paints and coatings. Its alleviation mechanisms include providing nutritional value, activation of oxidative stress system, and enhancement of plant’s physiological and biochemical functions. For example, seed soaking with ZnO NPs (50 mg/L) promoted glycolytic metabolism and cell wall biosynthesis, promoting the germination of corn seeds and the growth of roots and embryos [93]. The combined application of ZnO NPs (500 mg/kg) and arbuscular mycorrhiza (AM) significantly promoted soybean growth [94]. Applying ZnO NPs to wheat reduced the leaves’ electrolyte leakage (EL) rate and increased the antioxidant enzyme activity in the leaves [95]. ZnO NPs can relieve the drought stress to sorghum by increasing the absorption of nitrogen and potassium [96].

Ali et al. reported that combined application of ZnO NPs (foliar spraying) and biochar (soil application) effectively relieved the Cd stress, which was more efficient than the treatment of adding ZnO NPs or biochar separately [95,97,98]. It suggests that the combined application of NPs with other materials may be a promising approach in plant HM reduction.

Arsenic content in roots and shoots of rice was significantly reduced by both ZnO NPs and Zn^2+^ [99]. In contrast, Cd accumulation in rice shoots was only reduced by ZnO NPs, while Zn^2+^ increased the Cd accumulation in shoots. Khan et al. found that wheat biomass, yield and photosynthesis decreased under normal water conditions and Cd stress, while drought stress further enhanced the negative impact of Cd stress on wheat, while the addition of ZnO NPs alleviated both Cd and drought stress [100]. Hussain et al. applied different concentrations of ZnO NPs (25, 50 and 100 mg/L) to Cd-stressed wheat by foliar spray and soil application, and they found that both applications of ZnO NPs promoted wheat growth, photosynthesis and grain yield under Cd stress [101]. However, the foliar application was more efficient than soil application in increasing grain yield and reducing Cd content in grains.

Rizwan et al. soaked wheat seeds in ZnO NPs (25, 50, 75 and 100 mg/L) containing medium in the dark at 25 °C for 20 h, then selected seeds with similar germination to be sown in cadmium-contaminated soil [102]. Compared with the control group, the grain yield of wheat increased, the content of Cd in the grain decreased, the content of chlorophyll a and b, and SOD and POD activity of wheat increased significantly, and the electrolyte leakage rate decreased. The results showed that using an appropriate concentration of ZnO NPs to germinate wheat seeds can enhance wheat resistance to Cd.

### 3.3. Impacts of Non-Metal Based Nanoparticles Impact on the HM Stress in Plants

#### 3.3.1. Selenium

Selenium (Se) is an essential trace element for humans, which has many important biological functions, such as immune regulation, antioxidant, antiviral and anti-cancer properties [103,104,105]. Se is mainly supplemented by eating cereal crops, but in most rice-producing countries in Asia, the content is low. A recent survey shows that about 72% of areas in China are Se-deficient areas [106]. According to WHO, approximately 15% of the world’s population is deficient in Se, and China is one of the world’s 40 Se-deficient countries [107]. Therefore, Se containing fertilizer application to increase the Se content in cereal crops is essential for human health.

As an important active component of glutathione peroxidase, Se can regulate the activity of antioxidant enzymes, remove the ROS accumulated in plants, and thus effectively enhance the stress resistance of plants. For example, it has been found that the application of Se may reduce Cd uptake and toxicity in rice [108,109,110]. Recently, Huang et al. found that the inhibition effect of Se on Cd absorption of rice may be related to the formation of root surface iron film [111]. With the addition of Fe^2+^, Se promoted the formation of root surface iron film of rice and greatly reduced Cd accumulation in rice.

Zhang reported that Chinese cabbage’s biomass, height, leaf chlorophyll content, SOD, and GSH-Px increased and the Cd content and MDA of leaves decreased after foliar spraying of Se NPs (1.25 and 12.5 mg/L) to Chinese cabbage under Cd stress (10 and 20 mg/kg) [112]. Both in-soil (0.5 and 2.5 mg/kg) and foliar (0.5 and 1.0 mmol/L) application of Se NPs improved the physiological indices of rice in Cd-contaminated soil (total Cd 1.62 mg/kg), among which the foliar spraying had better effects on increasing the yield, relative content of chlorophyll in rice and reducing the content of Cd in rice than soil application [113]. In Cd and Pb co-contaminated soil, foliar spraying of Se NPs on rice effectively increased rice yield, biomass, protein content and Se content and reduced phytic acid production in brown rice [45]. It was also demonstrated that Se content and phytic acid are negatively correlated with Cd and Pb concentrations. Considering the safety factor, foliar spraying of Se NPs can significantly reduce the content of Cd in grains, making it more practical in actual production.

#### 3.3.2. Silicon

Silicon (Si) is the second most abundant element in the Earth’s crust after oxygen. Although it is not an essential element for higher plants, it is inextricably linked to plant growth, especially for Gramineae species (such as rice) [114]. Several studies have found that the application of Si can enhance plant resistance to abiotic stress [115,116]. At present, the widely recognized alleviation of HM stress by Si is through the following four mechanisms: 1) activation of the physiological and biochemical defense systems to enhance ROS scavenging [117], 2) immobilization and complexation of HM to reduce HM biological activity [118,119], 3) acting as a nutrient source and stimulating the production of structural protective agents chelated with HM [53], 4) regulation of the expression of HM transport genes, such as overexpression of vacuolar H^+^-pyrophosphatase1 (OVP1) [52].

Most of the early research has been carried out in the hydroponic conditions. For example, rice growing in the nutrient solution (20 μM CdCl_2_, foliar application of 2.5 mM nano Si) was shown to have smaller MDA and Cd content in the shoot, indicating that nano Si reduced the Cd stress [120]. In a rice cell culture experiment, the proportions of live cells in Cd solution (40 μM) were increased by 95.4%, 78.6% and 66.2% after addition of different size Si NPs (19, 48 and 202 nm: 1.0 mM), respectively [60]. This may be explained by the down-regulation of Cd uptake and transport (OsLCT1 and OsNramp5) genes and up-regulation of Cd transport (OsHMA3) and Si uptake (OsLsi1) genes. In composite stress conditions, Si NPs treatments improved wheat growth and alleviated Cd (bioavailable Cd 1.21 mg/kg) and drought (35% of soil water holding capacity) stress [121]. Hussain et al. reported that foliar application of Si NPs (10 and 20 mg/L) effectively decreased the accumulation of Cd in grains and increased the yield [45]. Besides, they demonstrated that combined application of Se NPs (20 mg/L) and Si NPs (10 mg/L) highly reduced Cd (62%) and Pb (52%) in rice grains.

Sousa et al. found that Si NPs (4 mg/kg) alleviated the toxicity of Al to maize by activating the antioxidant defense systems; at the same time, Si NPs did not influence the accumulation of Al in maize [122]. These results are contradictory with the above findings, and the reason may be related to different concentrations or types of HM used in these studies. In the field of combined application of NPs and microorganisms, Fatemi reported that Si NPs and Pb-resistant strains of the microbes more effectively alleviated the Pb toxicity to coriander than the single treatments of Si NPs or microbes [123].

#### 3.3.3. Hydroxyapatite

Phosphorus-containing amendments have been proven effective in the in-situ remediation of HM contaminated soils because they can release phosphates to form precipitates and complexes with HM in the soil [124]. Previous studies have shown that after adding hydroxyapatite (HAP) to the soil, it can release phosphate to form complexes with HM [125,126]. Also, the Ca ions on the surface of HAP could exchange with the HM ions in the soil to reduce the harm of HMto plants [127]. HAP with different particle sizes was proved to repair HM contaminated soil, for example, normal, micro, and nano HAP increased soil pH and decreased the amount of CaCl_2_-extractable, exchangeable Cu and Cd [56].

Furthermore, effects of HAP NPs (5, 10, 20 and 30g/kg) on *Brassica chinensis* L. under Cd stress (10 mg/kg) were studied in a pot experiment [128]. Compared with the control, the biomass, chlorophyll, vitamin C, and the activities of SOD, CAT, and POD in plant shoots were enhanced; Cd and MDA contents in the shoots were decreased. Sun et al. reported that the content of Cu and Zn in ryegrass significantly decreased by adding HAP NPs [129]. Xing investigated the soil properties and Cd, Cu, Zn and Pb content in soil and rice after addition of HAP NPs for one and three years [130]. They concluded that HAP NPs significantly decreased the Cd content in roots and grains both after one and three years. Compared with one year, the immobilization ability of HAP NPs for Pb was slightly increased after three years, while significantly decreased for Zn, Cd and Cu after three years.

The pH of the Cd-contaminated soil (10 mg/kg) increased after application of HAP NPs (0.2%, 0.5%, and 1%) and significantly increased the catalase and urease activities in the soil [131]. Besides, the plant height and biomass increased, and the Cd concentration in the edible part was reduced significantly, which shows that HAP NPs effectively alleviated the Cd stress. In Cd (37 mg/kg) and Pb (615 mg/kg) compound stress condition, the application of HAP significantly increased the soil pH, whereas the application of HAP NPs slightly reduced the soil pH, both of which significantly reduced the content of Diethylenetriaminepentaacetic acid (DTPA)-extracted Pb and Cd in soil [132]. Although HAP application reduced Cd absorption by the shoots and roots, the addition of HAP and HAP NPs both significantly increased the Cd content in tobacco leaves. It indicates that although NPs can reduce the content of HM in some parts, they may also increase it. Therefore, potential ecological risks and mechanisms still need further research.

## 4. Summary and Future Prospective

**Figure 4:**
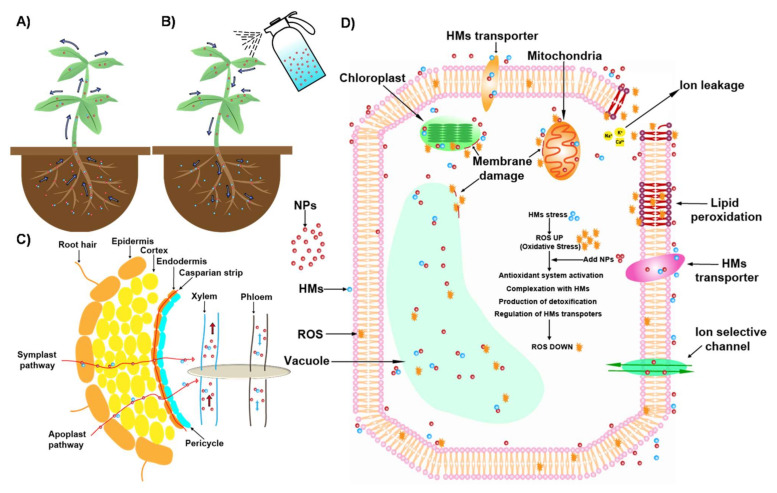
Schematic diagram of possible mechanisms of NPs to alleviate the HM stress in roots and cells. (**A**) Soil application of NPs to plant under HM stress. (**B**) Foliar spaying of NPs to plant under HM stress. (**C**) NPs enter plants possibly through symplast and apoplast. (**D**) Possible mechanisms of NPs to alleviate the damages caused by HM.

This review summarized the research progress in applying various NPs through seed priming, soil application, foliar spraying, and solution application to improve plant HM stress resistance. The morphology, physiology and biochemistry of plants are significantly affected by HM stress. Crucial strategies to improve plant HM resistance include reducing the concentration of bioavailable HM in the soil, regulating the expression of HM transport genes in plants, improving the ability of the apoplastic barrier to intercept HM, providing more nutrition to plant, enhancing the ability of plant antioxidant systems, improving the function of organs and enhancing the production of structural protective agents (such as root exudates, phytochelatin and organic acids). In addition, we summarized the effect of different NPs on the plants under Cd stress (Table 1) and a schematic diagram of possible mechanisms of NPs to alleviate the HM stress in roots and cells (Figure 4).

With the continuous advancement of science and technology, several types of NPs become available in the market including surface coated or blended with other materials (such as surfactants), which can enhance or enrich their existing functions. Moreover, physicochemical properties of NPs (size and charge) that determine their interaction with HM in soil in the presence of agricultural plants still need to be further studied. Because the porosity of plant cell walls is 15 nm, some NPs may penetrate the cell wall [61]. Besides, negatively charged NPs can penetrate the membrane more effectively than positive and neutral [133]. Although several studies have reported on the toxicity of NPs to animals and plants, most of them are in the presence of single NPs, and more in-depth studies on the toxicity of more complex environments are still needed.

In the research on alleviating HM stress, the exposure level of NPs and the different application methods affect plants differently. It is an urgent problem that low dosage has a non-significant control effect, and excessive dosage will increase unnecessary costs or harmful effects on plants. Besides, there are still ambiguities regarding NPs application methods in the soil environment. Therefore, it is necessary to study further the mechanisms and treatment effects of different application methods. Furthermore, the combined application of different kinds of NPs with other materials is undoubtedly a new research direction. For example, NPs application with other alleviators like hydrogen peroxide, nitric oxide, auxin, biochar, and HM-resistant strains of the microbes need to be investigated.

Big data and artificial intelligence (AI) technology are already being integrated into environmental science alone with other disciplines. For example, Rossi et al. used AI to predict the accumulation of CeO_2_ NPs and Cd in *Brassica napus* according to the physiological parameters of *B. napus* [134]. With experimental data accumulation and the development of technology, it is believed that various physiological parameters of plants and the content of HM in plants can be predicted based on the property of the soil, the variety of plants, and the property of NPs. It is possible to select appropriate types and concentrations of NPs for application, which would reduce unnecessary costs and maximize the beneficial effect.

Finally, the cost of NPs is one of the essential factors restricting their wider application in the agriculture sector. Therefore, synthesizing NPs efficiently, using green methods, and economically is an important part of nanotechnology’s future development. Researchers still need to conduct more in-depth research to understand how NPs can improve plant resistance, and it is necessary to study further the impact of NPs on terrestrial environment. Besides, the combination with computers and other disciplines will provide us with a more precise application of NPs and environmental impact to better promote the technology of NPs to control HM stress.

## Figures and Tables

**Table 1:** Effects of different NPs on the plants under Cd stress.

NPs	Plant	HM	Methods	Dose (mg/kg)	HM Content (%)	Biomass (%)	Details	Ref
Ce	Rice	Cd	Hydroponics	200	−7.04	14.29	Improved the rice growth indicators, photosynthesis and the level of 8-OHdG; did not affect the proline concentration; reduced the activities of SOD, POD and MDA.	[69]
	Soybean	Cd	Soil	500	7.18~23.20 (leaves)	−4.3~4.7 (dry weight)	Improved the Fv/Fm ratio and the Cd concentrations in leaves; significantly increased the accumulation of Ce in roots and older leaves.	[58]
Ti	Rice	Cd	Hydroponics	10~1000	−55.46~14.29 (leaves)	−12.77~8.14 (shoot)	Significantly increased net photosynthetic rate, chlorophyll and the activities of SOD and POD; reduced MDA and Cd accumulation in roots and leaves.	[81]
	Maize	Cd	Foliar	100~250	−17.80~15.20 (shoot)	0~11.03 (shoot)	Significantly decreased accumulation of Cd in shoots; enhanced the activities of SOD and GST; up-regulated metabolic pathways to alleviate Cd stress.	[46]
			Soil	100~250	−2.79~26.00 (shoot)	−46.08~17.65 (shoot)	Significantly decreased plant dry weight and chlorophyll; significantly increased Cd and Ti accumulation.	
Fe	Wheat	Cd	Foliar	5~20	−80.92~−22.75	26.96~72.35	Improved the plant growth indicators, photosynthesis and the activities of SOD and POD; decreased EL, MDA contents, and the accumulation of Cd in grains.	[89]
		Cd	Soil	5~20	−84.80~−23.20	30.23~93.36		
Zn	Wheat	Cd	Foliar	25~100	−77.00~−30.00	36.91~97.62	Increased the yield, photosynthetic rate and the activities of SOD and POD in leaves; decreased EL, MDA contents, and Cd accumulation in grains.	[101]
		Cd	Soil	25~100	−78.00~−16.00	27.71~74.24		
	Wheat	Cd	Soil (Seed priming)	25~100	−83.26~−30.47	24.83~74.81	Seed priming Significantly improved the wheat growth indicators, photosynthesis and the ac activities of SOD and POD; reduced the EL, MDA contents, and the accumulation of Cd in grains.	[102]
Se	*Brassica napus*	Cd	Foliar	1.5~12.5	−66.11~22.83	10.34~30.53	Increased the biomass, chlorophyll and activities of SOD and GSH-Px; but high dose promoted the accumulation of Cd in grains.	[112]
	Rice	Cd	Soil	0.5~2.5	−32.67~−17.33	0.40~18.62	Increased the biomass, stomatal conductance and chlorophyll; reduced the accumulation of Cd in grains and leaves and the exchangeable Cd in soil.	[113]
Si	Rice	Cd	Foliar	2.5 mM	−35.47~−16.88	11.49~45.67	Increased mineral elements content (Mg, Fe, and Zn), GSH content and activities of SOD and POD in shoots; reduced Cd contents and the activity of CAT in shoots.	[120]
	Wheat	Cd	Soil	25~100	−82.99~−22.01	25.23~74.10	Improved the plant growth indicators, photosynthesis and the activities of SOD and POD; reduced the H_2_O_2_, EL, MDA and Cd content.	[121]
HAP	Pak choi	Cd	Soil	5~30 g/kg	−62.36~−27.12	7.97~20.23	Improved the plant growth indicators, the level of chlorophyll and vitamin C, and the activities of SOD, CAT and POD; reduced MDA and the accumulation of Cd in leaves.	[128]

^1^ 8-OHdG: 8-hydroxy-2-deoxyguanosine. SOD: superoxide dismutase. POD: peroxidase. MDA: malondialdehyde. GST: glutathione S-transferase. EL: electrolyte leakage. GSH-Px: glutathione peroxidase. GSH: glutathione. CAT: catalase. Fv/Fm: maximal quantum yield of PS II.

## Data Availability

The data presented in this study are available in [this article].
